# Multitask machine learning models for predicting lipophilicity (logP) in the SAMPL7 challenge

**DOI:** 10.1007/s10822-021-00405-6

**Published:** 2021-07-17

**Authors:** Eelke B. Lenselink, Pieter F. W. Stouten

**Affiliations:** Galapagos NV, Generaal De Wittelaan L11 A3, 2800 Mechelen, Belgium

**Keywords:** D-MPNN, Multitask machine learning, logP prediction, SAMPL7

## Abstract

Accurate prediction of lipophilicity—logP—based on molecular structures is a well-established field. Predictions of logP are often used to drive forward drug discovery projects. Driven by the SAMPL7 challenge, in this manuscript we describe the steps that were taken to construct a novel machine learning model that can predict and generalize well. This model is based on the recently described Directed-Message Passing Neural Networks (D-MPNNs). Further enhancements included: both the inclusion of additional datasets from ChEMBL (RMSE improvement of 0.03), and the addition of helper tasks (RMSE improvement of 0.04). To the best of our knowledge, the concept of adding predictions from other models (Simulations Plus logP and logD@pH7.4, respectively) as helper tasks is novel and could be applied in a broader context. The final model that we constructed and used to participate in the challenge ranked 2/17 ranked submissions with an RMSE of 0.66, and an MAE of 0.48 (submission: Chemprop). On other datasets the model also works well, especially retrospectively applied to the SAMPL6 challenge where it would have ranked number one out of all submissions (RMSE of 0.35). Despite the fact that our model works well, we conclude with suggestions that are expected to improve the model even further.

## Introduction

Lipophilicity plays a key role in drug discovery, from an indirect role in ADME (absorption, distribution, metabolism, and excretion), to toxicity and potency [1, 2]. Typically, in drug discovery projects, lipophilicity predictions are used by the chemist to drive the chemistry forward, i.e. to balance lipophilicity and potency [2]. Often however, the predicted values of lipophilicity, or derivatives thereof (such as LLE) [3], are used as is, without considering the uncertainty or error in these predictions. Moreover, the performance of machine learning-based methods reported in the literature is typically an overestimation of the true performance of these models [4, 5], because historically they were based on a division into random training and test sets that were structurally not very different. Time- and scaffold-based splits would provide more realistic assessments of the expected performance [6, 7].

Nevertheless, there are many well-performing programs/models to estimate logP, and the reader is referred to Mannhold et al. [8] for a more detailed overview. Machine learning (ML) approaches that predict logP come in two flavors: additive and property-based. Additive models such as XlogP3 [9] assume additivity of logP for different atom (types), while property-based models such as Simulations Plus (hereafter referred to as S+) logP [10] use statistical methods and molecular descriptors. A third flavor of methods, exemplified by COSMOtherm [11] is physics-based rather than the result of an ML approach. It is also widely used although the calculation speed is usually about an order of magnitude lower than for ML models.

The SAMPL blind prediction challenges have been a way to quantitively benchmark different methods. For example, the recent SAMPL6 challenge for logP assessed 91 different prediction methods [12]. Motivated by this opportunity to prospectively assess our approach to constructing multitask ML models, we decided to participate in the SAMPL7 challenge [13].

Recent work in ML has revealed the advantages of neural networks, especially when employing techniques such as: (1) learned representations, and (2) multitasking.

1. Graph-based convolutional neural networks (GCNNs) have shown to hold promise (given sufficient data), ever since their application was first described [14]. For instance, weave, one type of GCNN, has been shown to prospectively outperform Random Forests (RFs) when applied to DNA-Encoded Library (DEL) screening data [15]. Recently, Directed-message passing neural networks (D-MPNNs) that are based on learned representations rather than fixed molecular descriptors have been introduced [6]. These D-MPNNs have shown to perform well across the board without (much) hyperparameter optimization. D-MPNNs iteratively generate representations of molecules by transmitting information across bonds (directed), in the message passing phase. Subsequently, in the readout phase, these representations are used to predict the property of interest. For further information refer to Yang et al. and Wu et al. [6, 16]. D-MPNNs have shown to outperform RFs and other GCNNs across the board [6]. Moreover, in practice, D-MPNNs have been applied successfully to the discovery of novel antibiotics [17].
2. Another advantage of neural networks is their ability to learn multiple tasks simultaneously. The concept of developing one model for multiple tasks has been first utilized in the Kaggle bioactivity challenge hosted by Merck, where the winning team used a mix of singletask and multitask neural networks [18]. Subsequently, the added value has been demonstrated with several different datasets [19], such as for modelling of ChEMBL bioactivity data [20], and for ADME modelling [21, 22]. Overall, multitask models are particularly beneficial if the tasks are related [18].

In this manuscript the steps that were undertaken that led to our final model are illustrated. Starting from a test set that is similar to the molecules of the SAMPL7 set, the following steps were performed, and their effects evaluated:

1. Using a default D-MPNN architecture, and adding rdkit descriptors
2. Adding extra datasets as separate tasks: ChEMBL/AstraZeneca deposited dataset
3. Running a hyperparameter optimization
4. Adding logP/logD7.4 (= logD@pH7.4) predictions (calculated with S+ ADMET Predictor V9.5 [10]), either as descriptors or as tasks.

Overall, those steps led to the final model that scored second (out of 17 ranked submissions) in the SAMPL7 LogP challenge.

## Methods

### Datasets and test set creation

Biovia’s Pipeline Pilot v17.2.0.1361 [23] was used for most of the data processing steps.

The first set of logP data was extracted from the Opera datasets [24], which contained 13,963 structure-logP datapoints.

Because we wanted to build a test set that mimicked the SAMPL7 challenge molecules, a tailored training/test set was created, as follows:

233 molecules were selected for the test set, based on their maximum similarity to the 22 SAMPL7 molecules [25] being greater than 0.25 (Tanimoto Coefficient (TC), ECFP_6 fingerprints [26], as implemented in Pipeline Pilot). To make the training/test split simultaneously more realistic and more challenging), the training set was constructed by taking the remainder of the molecules and filtering out 756 molecules with a similarity > 0.4 compared to the test set (TC, ECFP_6 fingerprints) and one molecule with an incorrect smiles code, leading to a training set of 12,973 molecules.

5539 additional datapoints were extracted from ChEMBL_25 [27], using logP as a query in the assay description. Calculated logP datapoints were discarded. For the logD7.4 data we used all data available in the AstraZeneca deposited set (DOC ID: CHEMBL3301361).

Finally for models 9, 10, and 12, S+ logP and logD7.4 were calculated for all molecules using ADMET Predictor V9.5 [10]. For model 10 we added S+ logP and logD7.4 as descriptors (in a separate input layer), while for model 9 those calculated properties were used as (helper) tasks. The model learns S+ logP and logD7.4 as additional tasks in the loss function on the basis of the structures. The use of these calculated properties as additional helper tasks could help regularize the model.

### D-MPNN training

Directed message passing neural networks [6] were trained on a workstation containing a NVIDIA RTX6000. Rdkit [28] in python was used to convert the molecule files (sdf) into a format compatible for use in chemprop (with columns for smiles, logP, logP_Chembl, logD7.4_AZ, etc.).

Because an external test set was used, the test set was omitted in the training loop of chemprop (–split_sizes 0.9 0.1 0)*.* A hyperparameter optimization run was performed using the hyperparameter_optimization.py script provided by chemprop, which uses hyperopt [29] to tune the hyperparameters of the neural network. The search grid was not changed, and a scaffold split (–split_type scaffold_balanced) was used for this evaluation. The settings that were used for the “optimized model” were: 5 message passing  steps (–depth 5), dropout of 0 percent (–dropout 0), 3 feed forward layers (–ffn_num_layers 3), and 700 neurons in the hidden layers (–hidden_size 700).

Confidence intervals on the performance metrics were calculated using sklearn [30] and bootstrapping, using mlxtend [31].

After having developed the series of models from 1 through 12 in order to optimize the settings, we built the best possible model (12_Full) by creating an emsemble of 10 individual models on the basis of all available data (Opera, ChEMBL and AZ), without using a separate test set. Predictions submitted to the challenge were done on the basis of this Model 12_Full, while the Standard Error in the Mean (SEM) of the prediction for each compound was estimated on the basis of the 10 individual model predictions. For benchmarking purposes, a singletask ensemble model consisting of 10 individual models (11_Full) was developed using all Opera data.

### Other software

The following additional tools were used throughout the work described in this manuscript: AlogP (through Biovia’s Pipeline Pilot) [32] and XlogP3 [9].

## Results

### Baseline performance

The starting point for the experiments was the tailored, SAMPL7-biased dataset (see methods). For lipophilicity data we resorted primarily to the Opera dataset [24], which was used in one of the best performing models for the SAMPL6 challenge [12].

In our first set of experiments (Table 1: Models 1–3) we studied the impact of varying the settings and adding extra descriptors natively available in chemprop. “Out of the box” (default) the D-MPNN model already provided comparable performance to commercial solutions (RMSE model 1 = 0.45; RMSE S+ logP = 0.40). Having no knowledge of and no control over the training/test sets used to develop the commercial logP predictors, the comparison was primarily done to establish a baseline for further experiments.

**Table 1:** Overview of the optimization done on the model, performance (R^2^, RMSE, Spearman ρ) on the test set constructed for this challenge

Model	Description	R^2^	RMSE	Spearman ρ
–	AlogP	0.83 [0.71,0.90]	0.73 [0.55,0.93]	0.90 [0.85,0.94]
–	XlogP3	0.85 [0.75,0.92]	0.67 [0.48,0.87]	0.91 [0.87,0.95]
–	S+ logP	0.95 [0.91,0.97]	0.40 [0.32,0.48]	0.97 [0.94,0.98]
1	default	0.93 [0.89,0.96]	0.45 [0.36,0.57]	0.96 [0.94,0.97]
2	1 + rdkit	0.93 [0.89,0.96]	0.45 [0.37,0.55]	0.96 [0.94,0.98]
3	rdkit only	0.88 [0.82,0.92]	0.60 [0.50,0.70]	0.94 [0.91,0.96]
4	1 + ChEMBL merged	0.88 [0.81,0.92]	0.60 [0.51,0.71]	0.94 [0.92,0.96]
5	1 + ChEMBL separate	0.93 [0.88,0.95]	0.47 [0.38,0.58]	0.96 [0.94,0.98]
6	5 + AZ_logD7.4	0.94 [0.91,0.96]	0.42 [0.35,0.50]	0.97 [0.95,0.97]
7	5 + AZ_ADME	0.94 [0.90,0.96]	0.44 [0.36,0.51]	0.97 [0.95,0.98]
8	6 + hyperopt parameters	0.93 [0.88,0.95]	0.47 [0.39,0.58]	0.96 [0.94,0.97]
9	6 + S+ logP/logD7.4 as tasks	0.95 [0.93,0.97]	0.38 [0.32,0.44]	0.97 [0.96,0.98]
10	6 + S+ logP/logD7.4 as descriptors	0.95 [0.92,0.97]	0.39 [0.34,0.44]	0.97 [0.96,0.98]
11	1, ensemble of 10	0.94 [0.89,0.96]	0.44 [0.35,0.55]	0.96 [0.94,0.98]
12	9, ensemble of 10	0.95 [0.92,0.97]	0.39 [0.33,0.46]	0.97 [0.96,0.98]

The ordinal model numbers in the left-most column indicate the sequence in which the models were developed: for example model 6 (5 + AZ_logD7.4) means that the settings/data of model 5 were used and the AZ_logD7.4 data were added. The 95% confidence interval for the different performance metrics is shown between square brackets

Adding extra rdkit descriptors (calculated by descriptastorus, a library included in the chemprop package) as a separate layer (model 2) didn’t improve the performance on this test set, and came at an extra computational cost. To analyze the relative contributions of the learned representation and the molecular descriptors, respectively, we also trained a network only based on rdkit descriptors (model 3). As expected, this model performed substantially worse (RMSE model 3 = 0.60; RMSE model 1 = 0.45).

### Adding datasets as separate endpoints

To further improve the model performance and generalizability, two data sets were added to the Opera set (see methods):

1. Experimental logP data from ChEMBL,
2. DMPK/Physchem data, deposited by AstraZeneca.

Here we were interested in observing in which way additional data could help to improve the model. Separating the data for the two different endpoints (i.e. logP_Opera, logP_ChEMBL) outperformed aggregating all public data in one endpoint (i.e. logP_Opera + ChEMBL), but there is no substantial difference between model 5 and the default model (RMSE model 5 = 0.47; RMSE model 1 = 0.45). Expecting that generalizability will improve with more, chemically different data sets, subsequent models were developed with all data used to develop model 5.

The next two models (6 and 7) were developed with ChEMBL data from AstraZeneca for endpoints that may be correlated with logP: plasma protein binding, kinetic solubility@pH7.4, logD7.4, and intrinsic (microsomal and hepatocyte) clearance across species. These endpoints were used as “helper tasks.” In model 6 we only added the logD7.4 data, while in model 7 we added all data for the 4 endpoints. This was done to study the effect of including possibly less related tasks. Both models performed comparably and clearly better than model 5, proving that adding tasks for related properties is beneficial. Model 6 is somewhat better than model 7 (RMSE model 7 = 0.44; RMSE model 6 = 0.42), indicating that adding less related tasks does not provide any benefit. Subsequent models were developed with all data used to develop model 6. With model 8, we tried to improve on model 6 by tuning the hyperparameters (doing a run of hyperopt; refer to methods), and using the best settings. Tuning did not improve the model performance for this data set, however. Further work would be needed to assess whether other settings work better.

### Using predictions from other models as molecular descriptors or as “helper tasks”

Because adding AstraZeneca logD7.4 data led to increased performance, we decided to study the effect of adding calculated helper tasks. For this, predictions made by S+ logP and S+ logD7.4 were added. These models were chosen for several reasons:

First, because of S+’s excellent neural network-based pK_a_ models, which were developed with over 25,000 datapoints [33], and contribute to the accuracy of their logD7.4 model. Second, perhaps more importantly, because it could help regularize the model, when those properties are added as tasks (i.e. by learning the relationship between logP and logD). Based on this we hypothesized that adding both S+ logP and logD7.4 predictions could help improve performance.

Indeed, as shown in Table 1, adding S+ logP and S+ logD7.4 either as “helper tasks” (model 9) or as molecular descriptors (model 10) improves the performance of the model. One significant practical benefit of adding logP and logD7.4 as helper tasks rather than as descriptors, however, is that the helper tasks are only necessary to optimize the neural network (to make the model more robust and generalizable), but are not used to make actual model predictions. For that reason, slow models used as helper tasks can be tolerated.

**Fig. 1:**
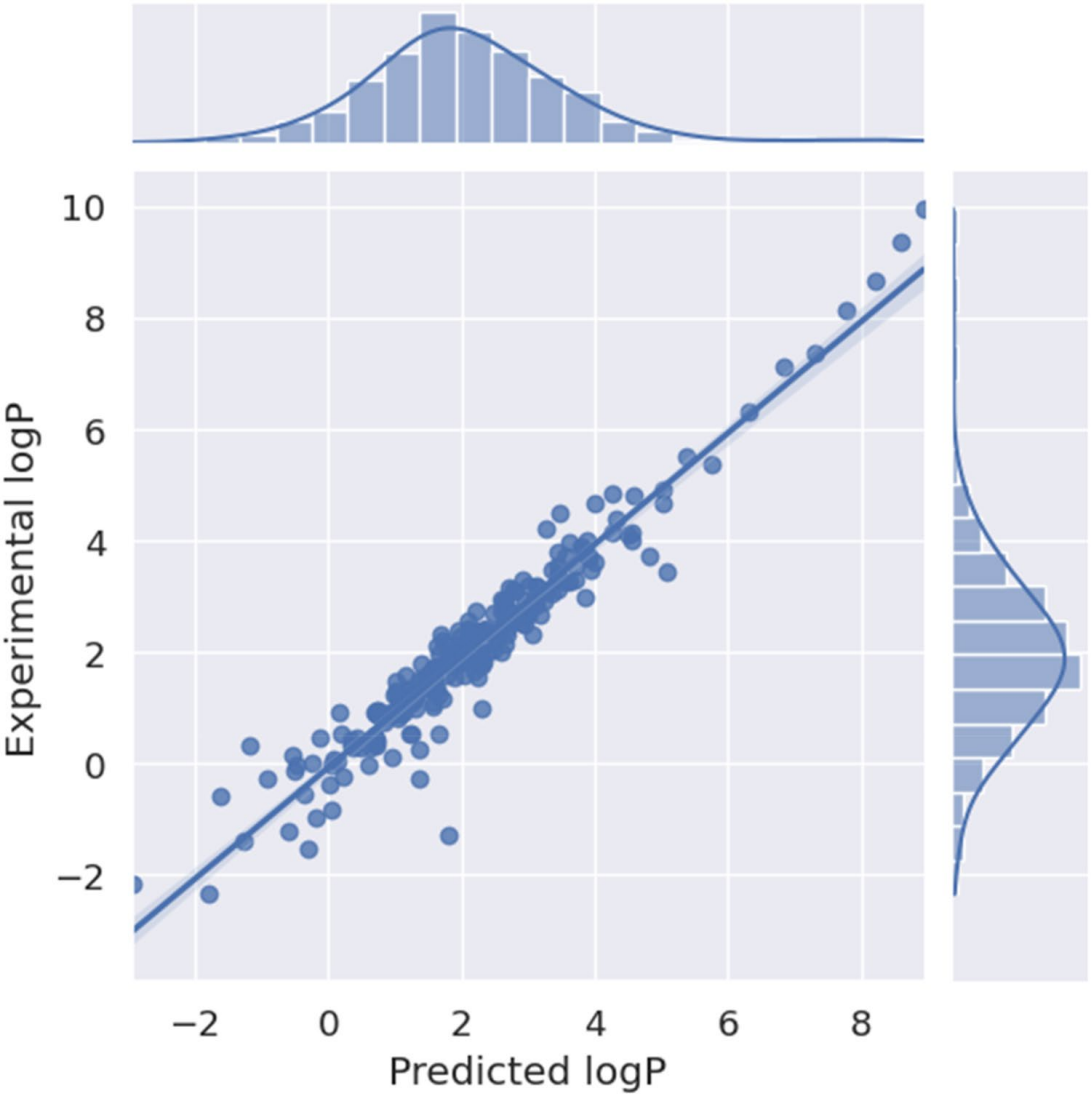
Scatter plot of the performance of the final model (Experimental log P versus Predicted logP) on the test set. On the top a distribution histogram of the predictions is shown and on the right a distribution histogram of the experimental values. The shaded area (very close to the identity line) represents the 95% confidence interval for the regression estimate

Finally, we compared the performance of multitask ensemble model 12 (model 9, but an ensemble of 10) with equivalent singletask ensemble model 11 (default model 1, but an ensemble of 10). The former model performed better, illustrating that the helper tasks and additional data did have a positive impact on the test set performance. The final performance of the model on the test set is shown in Fig. 1.

### Performance of the final full model on external sets

**Fig. 2:**
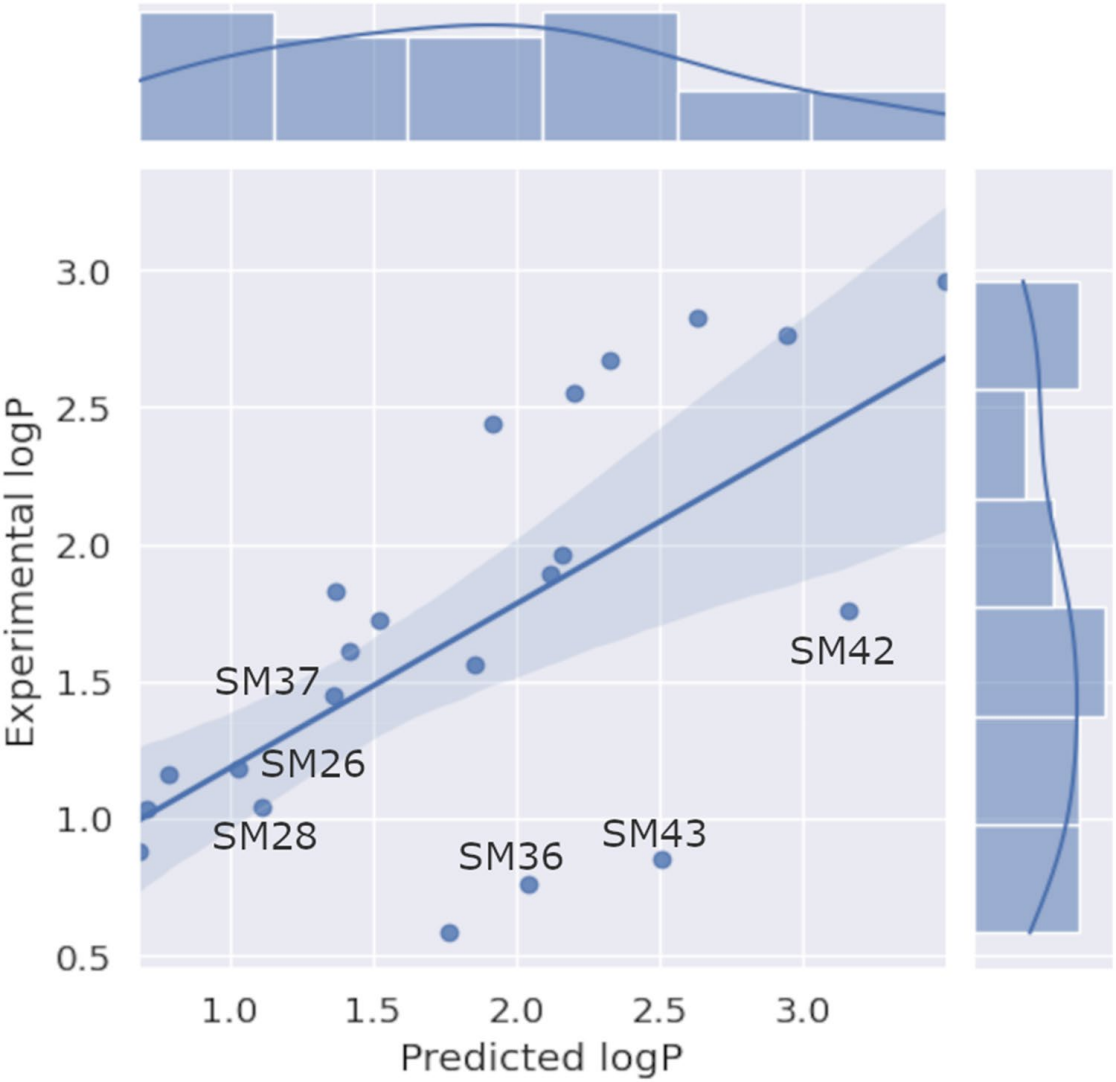
Scatter plot of the performance of the final model (Experimental log P versus Predicted logP) on the SAMPL7 molecules. The compounds discussed in the text and shown in Table 3 are labeled. On the top a distribution histogram of the predictions is shown and on the right a distribution histogram of the experimental values. The shaded area represents the 95% confidence interval for the regression estimate

Our primary goal was to develop a model that would be both generally applicable and perform well in the SAMPL7 challenge. As indicated in Methods, we developed two models on the basis of all available data (without having separate training and test sets): multitask ensemble model 12_Full and—for comparison—singletask ensemble model 11_Full. The performance of model 12_Full on the SAMPL7 compounds is shown in Fig. 2. Compared to other SAMPL7 competitors, our final multitask model (12_Full) performs very well: it ranked 2nd out of 17 ranked submissions, and 4th out of all 36 submissions. To assess the general applicability of our method we further benchmarked model 12_Full by applying it to several external data sets. Results for the SAMPL6 [12], SAMPL7, and Martel et al. [4] data sets are shown in Table 2. To verify that our observations regarding the benefits of adding extra data and using helper tasks were not limited to one data set, we also applied singletask model 11_Full to these external data sets. In addition, we established a baseline by applying the commercial models (AlogP, XlogP3 and S+ logP).

**Table 2:** Overview of the performance of the final multitask ensemble model (12_Full), used for the challenge, the singletask ensemble model (11_Full), and several commercial logP prediction tools on the SAMPL7, SAMPL6 and Martel et al. data sets [4]

Method	Dataset	R^2^	RMSE	Spearman ρ
AlogP	SAMPL7	− 0.30 [− 1.78,0.34]	0.82 [0.59,1.01]	0.42 [− 0.09,0.73]
XlogP3	SAMPL7	0.01 [− 1.12,0.46]	0.72 [0.55,0.87]	0.52 [0.07,0.78]
S+ logP	SAMPL7	0.06 [− 1.23,0.64]	0.70 [0.41,0.93]	0.62 [0.19,0.87]
Model 11_Full	SAMPL7	− 0.17 [− 1.49,0.38]	0.78 [0.52,1.01]	0.60 [0.13,0.86]
Model 12_Full	SAMPL7	0.17 [− 0.95,0.65]	0.66 [0.40,0.89]	0.63 [0.20,0.91]
AlogP	SAMPL6	0.56 [− 0.73,0.84]	0.44 [0.25,0.62]	0.83 [0.32,0.97]
XlogP3	SAMPL6	0.54 [− 0.69,0.82]	0.45 [0.29,0.58]	0.71 [0.05,0.94]
S+ logP	SAMPL6	0.42 [− 1.17,0.80]	0.51 [0.32,0.65]	0.71 [0.03,0.94]
Model 11_Full	SAMPL6	0.71 [− 0.25,0.90]	0.36 [0.24,0.46]	0.85 [0.40,0.99]
Model 12_Full	SAMPL6	0.75 [− 0.08,0.93]	0.34 [0.17,0.46]	0.82 [0.30,0.99]
AlogP	Martel et al.	− 0.15 [− 0.34,− 0.00]	1.27 [1.19,1.34]	0.73 [0.69,0.76]
XlogP3	Martel et al.	0.04 [− 0.11,0.16]	1.16 [1.10,1.21]	0.78 [0.75,0.81]
S+ logP	Martel et al.	− 0.26 [− 0.45,− 0.10]	1.33 [1.26,1.39]	0.71 [0.67,0.75]
Model 11_Full	Martel et al.	− 0.33 [− 0.51,− 0.18]	1.36 [1.31,1.41]	0.74 [0.70,0.77]
Model 12_Full	Martel et al.	− 0.00 [− 0.14,0.12]	1.18 [1.13,1.23]	0.76 [0.73,0.80]

The 95% confidence interval for the different performance metrics is shown between square brackets.

On both the SAMPL6 challenge and SAMPL7 challenge data sets multitask model 12_Full performed better than all other models we compared it to (singletask model 11_Full, AlogP, XlogP3 and S+ logP). In fact, for the SAMPL6 compound set, retrospectively analyzed, model 12_Full would have ranked number one, with an RMSE of 0.34, outperforming other methods like cosmotherm_FINE19 (RMSE: 0.38), and the global Xgboost-based QSPR model (RMSE: 0.39) [12].

For the Martel data set model 12_Full ranked second, close in performance to XlogP3. This dataset has been described as a challenging dataset, and in terms of absolute R^2^ and RMSE values, none of the five models perform adequately. More work would be needed to understand the poor performance of all models on the Martel data set, but that is beyond the scope of this paper.

In all cases the multitask model (12_Full) outperformed the singletask model (11_Full), although even the latter would have ranked a respectable 11/36 in the SAMPL7 challenge (considering all submissions).

### Analysis of predictions in terms of structures

To further investigate in which cases model 12_Full performs well, and in which cases it does less well, we analyzed the three best and three worst predictions, respectively, for the SAMPL7 challenge, and compared model 12_Full to two other high-ranking methods from the SAMPL7 challenge (Table 3). Both overpredicted compounds, SM42 and SM43, contain the same substructure, but the shift between the two was well-predicted (i.e. ΔlogP(phenyl → *N*-dimethyl is 0.91 experimentally and 0.66 predicted by model 12_Full). This suggests that our model 12_Full overestimates the lipophilicity of the phenyl-isoxazole-sulfonamide moiety. Both SM42 and SM43 were well predicted by TFE MLR (ranked first in the SAMPL7 challenge), which is a multiple linear regression model trained on a set of 82 druglike molecules (60 molecules containing sulfonamides) [34], indicating that for this particular moiety a more general model like ours does not perform as well as a tailor-made model. COSMO-RS [11, 35] exhibited the same behavior as our model, overpredicting both SM42 and SM43.

**Table 3:** The top three compounds in terms of largest error (SM43, SM42 and SM36) and lowest error (SM26, SM37 and SM28) for model 12_Full

Structure	ID	Experimental	Model _Full	TFE MLR	COSMO-RS
	SM43	0.85 ± 0.01	2.51 ± 0.10	0.38	2.59
	SM42	1.76 ± 0.03	3.16 ± 0.05	1.57	3.48
	SM36	0.76 ± 0.05	2.05 ± 0.10	2.63	2.29
	SM37	1.45 ± 0.10	1.36 ± 0.11	1.44	1.72
	SM26	1.04 ± 0.01	1.11 ± 0.06	1.18	1.22
	SM28	1.18 ± 0.08	1.03 ± 0.06	1.87	0.65

The SEMs for both the experimental data and the predictions by model 12_Full are given behind the ± sign. Results from two other methods (one statistical, one physical) that participated in the challenge, TFE MLR and COSMO-RS, are shown as a reference [34, 35]

Perhaps more puzzling is that model 12_Full, COSMO-RS, and TFE MLR overpredict SM36, while they all correctly predict SM37. This is a similar transformation as SM42 to SM43 (phenyl → *N*-dimethyl). In this case, however, the phenyl group has been experimentally determined to be less lipophilic than the *N*-dimethyl moiety (ΔlogP(phenyl → *N*-dimethyl) is -0.69 experimentally and 0.69 predicted by model 12_Full). Generally, a phenyl group is more lipophilic than a *N*-dimethyl moiety, but this is not observed for the latter case. More work would be needed to understand this puzzling outlier.

## Discussion and outlook

This manuscript serves as a walkthrough for the steps that were taken to develop an optimal model, with which to participate in the SAMPL7 challenge. Overall, the model that we developed performs very well, although in most cases only incremental improvements between subsequent models were observed.

Additional combinations can be tested to improve the model performance even more: using additional data (i.e. Martel’s) and/or predictions by other models (e.g. XlogP3) may well impact the model performance favorably. Although such a comprehensive investigation is beyond the scope of this paper, it may well be the topic of future work of ours.

In and by itself, the concept of modelling multiple properties as separate tasks within a multitask approach is not novel. What is novel, however, is that here we also consider different datasets for the ***same*** property as different tasks (e.g. ChEMBL_logP and Opera_logP). For many other types of assays this kind of data separation should make sense, too: e.g. shake-flask versus chromatographic logD, thermodynamic versus kinetic solubility, and functional versus binding assays.

Training a logP model on the basis of multiple predicted values is not novel per se: this has e.g. been done by JPlogP [36]. However, to the best of our knowledge, the addition of calculated properties as helper tasks to a multitask model is novel, and we expect it to have a wider applicability. Since these tasks only need to be calculated once, comprehensive further studies can easily and should be done to investigate whether the models improve if a larger set of (diverse) predictors were added as helper tasks.

An additional area of improvement could be the inclusion of physics-based features/predictions. ML models based on QM-derived features, such as ANI [37], allow for rapid estimation of QM-derived features. For ADME modelling QM-derived features have indeed improved model performance. Rather than going the QM-ML route, however, one could use other physics-based predictions that provide accurate logP estimates as additional tasks [38] in the same vein as we describe in this paper.

Finally, one improvement that is absolutely essential (not only for our models) is the proper estimation of the uncertainty of the predictions. Recently bayesian-based approaches have been described [39], also e.g. complementary to D-MPNN [40]. If uncertainties of the predictions could be accurately estimated this would impact in several ways: for instance to decide which compounds need to be made/tested in drug discovery projects, but also to decide which compounds need to be made/tested in order to improve the model.

## Conclusions

In this manuscript we have discussed the steps that were taken to create an optimal D-MPNN based logP prediction model. This model was constructed for the SAMPL7 challenge, where it scored 2/17 in ranked submissions, and 4/36 in all submissions. Three key improvements over the default D-MPNN model were the result of using: (1) additional data sets for the same and related properties as helper tasks, (2) predicted properties (S+ logP/logD7.4) as helper tasks, and (3) an ensemble of models. In a retrospective analysis the model also outperformed other methods when applied to the compounds from the previous SAMPL6 challenge. Performance was second of the methods applied to the Martel data set, but not very good in absolute terms, indicating that further work is warranted. Based on our results, we are convinced that ensembles of multitask models, developed with helper tasks and employing predictions by other models for related properties have great potential application well beyond modelling logP.

## Data Availability

The data used in training the final model, and final models will be shared on github: https://github.com/lenselinkbart/SAMPL7_paper.
